# Internal operations in the hippocampus: single cell and ensemble temporal coding

**DOI:** 10.3389/fnsys.2013.00046

**Published:** 2013-08-29

**Authors:** George Dragoi

**Affiliations:** The Picower Institute for Learning and Memory, RIKEN-MIT Center for Neural Circuit Genetics, Department of Biology and Department of Cognitive Sciences, Massachusetts Institute of TechnologyCambridge, MA, USA

**Keywords:** learning and memory, hippocampus, phase precession, cell assemblies, theta sequences, preplay, replay

Most of our cognitive life depends on our brain's ability to generate internal representations of the external world. The hippocampus is a brain structure that supports the formation of internal representations of the spatial environment (O'Keefe and Nadel, 1978) as well as the formation (Scoville and Milner, 1957) and consolidation (Squire and Alvarez, 1995) of episodic memories. In rodents, hippocampal pyramidal cells are active at discrete places along the trajectory of the animal in linear and two-dimensional spatial environments, and therefore are called place cells (O'Keefe and Dostrovsky, 1971). During exploratory behavior, the firing rates of individual place cells are thought to encode the moment-to-moment location of the animal in space (O'Keefe and Dostrovsky, 1971; Wilson and McNaughton, 1993). With reference to the background local field potential theta oscillation (~8 Hz), individual place cells oscillate at slightly faster frequency (~10 Hz) and fire at more advanced theta phases the further the animal travels through the cell's place field, a phenomenon called phase precession (O'Keefe and Recce, 1993; Skaggs et al., 1996; Huxter et al., 2008). Since most place cells go through almost a full 360° cycle of precession from the beginning to the end of their place field (O'Keefe and Recce, 1993), the theta phase of firing is thought to encode the distance of the animal relative to the beginning of the place field (Huxter et al., 2003).

About half of the pyramidal cells that are simultaneously recorded from the CA1 area of the rodent hippocampus display a place field in a given environment (Wilson and McNaughton, 1993). This implies that an individual cell will have similar place field activities (rate and phase) in multiple environments and that, alone, its activity is not sufficient to unambiguously represent or recall a specific spatial experience. Furthermore, within the same spatial environment, groups of place cells can be part of different neuronal ensembles (Wood et al., 2000; Pastalkova et al., 2008) that can flicker between distinct representations across theta cycles (Kelemen and Fenton, 2010; Jezek et al., 2011; Dupret et al., 2013). Consequently, when it comes to internal spatial representation and episodic memory formation, the activity of place cells must be taken into consideration at the ensemble level. As rodents engage in running along a linear or two-dimensional spatial environment, a sequence of place cells is activated according to the location of their place fields (Nadasdy et al., 1999; Lee and Wilson, 2002; Dupret et al., 2010; Pfeiffer and Foster, 2013). Moreover, within each theta cycle, sequences of place cells with partially overlapping place fields fire with compressed temporal delays and in a temporal order that correspond to the distance (Dragoi and Buzsaki, 2006) and the order (Skaggs et al., 1996; Lee and Wilson, 2002) between the location of their place fields along the linear trajectory, respectively. This phenomenon is known as temporal compression (Skaggs et al., 1996) of spatial sequences during theta or, simply, theta sequence compression (Dragoi and Buzsaki, 2006). The processes of phase precession and theta sequence compression are considered to be the manifestation of two aspects of phase coding of spatial information in the hippocampus, one at the single neuron level (Jensen and Lisman, 2000) and the other at the neuronal ensemble level (Dragoi and Buzsaki, 2006).

One prevalent view regarding the role of the hippocampus in the encoding of spatial information posits that during a novel spatial experience, ensembles of sequential place cells are independently driven by the external stimuli specific to the experience and their stimulus driven theta phase precession results in the first time expression of specific, compressed temporal firing sequences across the ensemble (Skaggs et al., 1996; Lee and Wilson, 2002). If indeed the neuronal ensemble encoding of the novel spatial experience and the neuronal organization in temporal and place sequences result exclusively or primarily from the stimulus driven theta phase precession of independent novel place cells during the experience, then the following predictions must also be met: (i) the strength of theta sequence compression should be correlated with the strength in theta phase precession; (ii) the expression of temporal sequences of neuronal firing should depend on the presence of theta oscillation in the hippocampus; (iii) the temporal sequences relevant to the encoding of a novel spatial experience should be created during the experience and should not be present before the animal's encounter with the new space. In this study, I aim to demonstrate that these predictions are not supported by the existing experimental data, a situation that invalidates the above view that led to their proposal. I will instead provide an alternative explanation for the temporal and place cell sequences expressed during the encoding of novel spatial experiences.

According to the first prediction, drastic changes in theta phase precession of multiple single place cells should have dramatic effects on theta sequence compression at the neuronal ensemble level. Theta phase precession and theta sequence compression are not homogeneous processes; they are more robust on the ascending portion of the place fields (Skaggs et al., 1996; Huxter et al., 2003) and weaker on the descending portion where spiking activity is noisier and assumes a relatively larger range of theta phases (Dragoi and Buzsaki, 2006). We (Dragoi and Buzsaki, 2006) and other groups (Foster and Wilson, 2007) artificially jittered the time of the spikes emitted on the ascending portion of the place fields and consequently morphed their phase precession to appear just like the one of spikes emitted in the corresponding descending portion of the fields. In spite of the phase precession becoming a homogenous process throughout the place field, the theta sequence compression of the jittered spikes remained heterogeneous, significantly stronger in the ascending portion of the place fields (Dragoi and Buzsaki, 2006). This finding indicates that theta phase precession in multiple single neurons is not simply generating theta sequence compression in the ensemble of place cells from the CA1 area of the hippocampus. Instead, theta sequence compression seems to reflect the more robust coordinated oscillation of sequential cellular assemblies (Hebb, 1949) in a theta frequency band (~10 Hz) that is faster (O'Keefe and Recce, 1993; Dragoi and Buzsaki, 2006) than the one of the field theta (~8 Hz). Temporal coordination of neurons and theta sequence compression cannot be simply explained by independently phase precessing cells (Dragoi and Buzsaki, 2006; Foster and Wilson, 2007), but rather rely on a transient increase in precise timing within and across sequential cellular assemblies.

During animals' exploratory states which are associated with theta oscillation in the hippocampus, sequential place cells fire in temporal sequences that are compressed 8–16 times (Skaggs et al., 1996; Dragoi and Buzsaki, 2006), depending on the spatial distance between their place fields. According to the second prediction, compressed temporal sequences of place cell firing depending on phase precession should not be expressed during epochs when theta oscillation is absent. However, very similar patterns of compressed temporal sequences of firing of place cells occur at a similar or slightly higher compression ratio during the following sleep (Nadasdy et al., 1999; Lee and Wilson, 2002; Ji and Wilson, 2007) or rest (Foster and Wilson, 2006; Diba and Buzsaki, 2007; Davidson et al., 2009; Karlsson and Frank, 2009), preferentially during sharp-wave ripple epochs, in the absence of theta oscillation in the hippocampus. The ripple-associated temporal sequences were believed to be the expression of a reactivation or replay (Buzsaki, 1989; Pavlides and Winson, 1989; Wilson and McNaughton, 1994; Lee and Wilson, 2002) of the previous activity during run, a process facilitated by an increase for several hours in the post-experience excitability of the previously active place cells (Battaglia et al., 2005). This indicates once again that the expression of temporal sequences of place cell firing does not depend on theta phase precession, but rather reflects the more general organization of neurons into coordinated sequential cellular assemblies (Hebb, 1949; Dragoi and Buzsaki, 2006).

**Figure 1:**
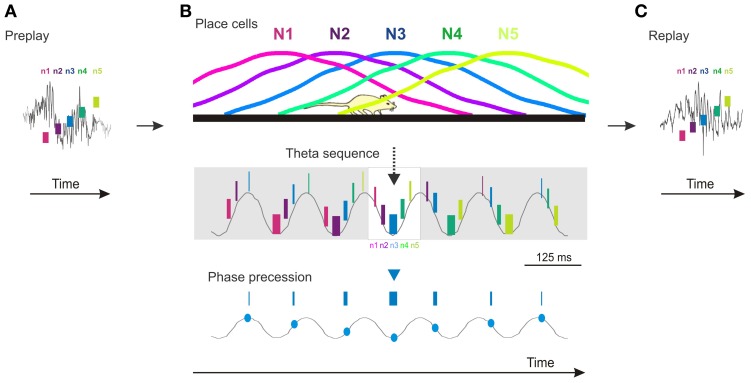
**Cartoon model of preplay, theta sequences, phase precession, and replay in the CA1 area of the hippocampus. (A)** Preplay of a future novel place cell sequence during a ripple event (gray line) occurring during sleep/rest (n1–n5, five neurons firing in temporal order; short vertical bars denote relative firing of neurons in time). **(B)** Top, place cell sequence of the five neurons (N1–N5) in **(A)** firing in the same order in space during run on a novel track. Middle, temporal sequences of firing of the five neurons (n1–n5) during theta oscillation (gray line) during the run. Neurons are color coded; the thickness of the short vertical bars denote relative firing rate, highest on the theta troughs. The sequence represented in the white rectangle in the middle matches the one during the ripple in **(A)** and the place cell sequence on the Top panel. Bottom, phase precession of place cell N3. **(C)** Replay of the place cell sequence (N1–N5) during a ripple event occurring during the sleep/rest following the run in **(B)**. The temporal sequences (n1–n5) in **(A)** and **(C)** perfectly matching the spatial sequences (N1–N5) in **(B)** occur in a subset of pre/replay events. Overall, pre/replay sequences are significantly correlated with the place cell sequences to an average absolute correlation value of ~0.75. Time scale bar in **(B)** also applies to **(A)** and **(C)**. All spikes **(A–C)** and their relation to theta oscillation and ripples are illustrations. Ripple and theta oscillation examples are adapted from experimental data.

The presence of temporal sequences during sharp-wave ripple epochs in the absence of theta oscillation (and phase precession) could suggest that once sequentially active cell assemblies are bound into a temporal sequence during theta they no longer need the theta oscillation for them to be expressed at a later time. This scenario would be consistent with the third prediction that posits that compressed temporal sequences of novel place cells should not be expressed before the novel spatial experience. However, temporal firing sequences reflecting the future order of place cell firing and future novel trajectories can be expressed during sharp-wave ripple epochs occurring during sleep or rest in naïve animals before they had any experience on long linear tracks (Dragoi and Tonegawa, 2011, 2013). This phenomenon called preplay (Dragoi and Tonegawa, 2011) demonstrates that Hebbian phase sequences (Hebb, 1949) occur in naïve animals and can precede the expression of structured novel place cell sequences (Figure 1A). The existence of preplay indicates that temporal sequences of place cells are not necessarily caused by an ongoing external input-driven theta phase precession, but rather represent the default mode of internal organization of the hippocampal network in sequential cellular assemblies. In this context, theta phase precession in multiple individual place cells is the expression of this oscillatory network organization at the single cell level (Figure 1B) aligned in phase to the intracellular subthreshold oscillations (Harvey et al., 2009). Consequently, the expression of theta sequence compression during encoding of a novel spatial experience is due in part to a rapid assignment of a subset of the existing motifs of temporal firing sequences to the novel experience (Dragoi and Tonegawa, 2013) in the form of novel place cell sequences. The allocation of place cells to novel spatial locations is followed by a rapid increase in their place field tuning, coordination, and stability mediated by synaptic plasticity mechanisms (McHugh et al., 1996; Kentros et al., 1998). The role of the external input appears to be primarily in the selection of the subset of temporal firing sequences from a larger pre-existing repertoire rather than in the de novo creation of the temporal sequences (Dragoi and Tonegawa, 2013). The place cell sequences are replayed during the following sleep/rest session (Figure 1C).

We explored the role of synaptic plasticity of intrinsic hippocampal circuitry in the internal organization of the hippocampal network in sequential cellular assemblies (Dragoi et al., 2003). If temporal firing sequences in the CA1 would simply be conveyed from the entorhinal cortex carrying spatial information about the external environment (Frank et al., 2000; Fyhn et al., 2004) then plastic changes in the CA3-CA1 circuitry should have a minimal effect on their expression. Induction of long-term potentiation (Bliss and Lomo, 1973) in the intra-hippocampal synaptic weight matrix resulted in novel place cell sequences being expressed in the CA1 area during the exploration of the familiar environment despite any changes in the external environment (Dragoi et al., 2003). New sequences of place fields were created at the expense of old sequences disappearing in the absence of any alteration in the global level of hippocampal excitability and overall place field features (Dragoi et al., 2003). The artificially-induced synaptic plasticity altered the place cell sequences, but this change was not induced by the cues from the external environment as they were kept constant. This result indicates that synaptic plasticity of intrinsic hippocampal connectivity plays a crucial role in assembling sequences of place cells whose compressed firing activity is subsequently associated (Dragoi and Tonegawa, 2011) with particular spatial experiences. Synaptic plasticity during the run experience plays a role in the additional spatial tuning and firing rate change of individual place cells particularly in the de novo exposures to novel tracks (Dragoi and Tonegawa, 2011), and appears to be complementary to the pre-existing synaptic structure in establishing the stable order of place cell firing.

The existence of preconfigured cellular assemblies and the phenomenon of preplay lead to a novel concept that an animal's encounter with a novel spatial experience is encoded in the hippocampus, in part using blocks of pre-made cellular firing sequences rather than creating all the sequences de novo in response to the external cues. This mechanism may contribute to the role of the hippocampus in prospective coding (Schacter et al., 2008), rapid learning (Tse et al., 2007), and imagining (Hassabis et al., 2007).
